# Supplementary material for: Rabies virus large protein-derived T-cell immunogen facilitates rapid viral clearance and enhances protection against lethal challenge in mice
Source: Commun Med (Lond). 2025 Apr 18;5:127. doi: 10.1038/s43856-025-00851-5 (PMC12008279; doi:10.1038/s43856-025-00851-5)
Supplement: Supplementary file 2 — Supplementary Information [file 43856_2025_851_MOESM2_ESM.pdf]

Rabies virus Large Protein-derived T-cell immunogen facilitates rapid viral clearance and enhances protection against lethal challenge in mice

Shimeng Bai[1,3†], Xinghao Pan[2†], Tianhan Yang[2†], Nan Gao[2], Cuisong Zhu[2], Ai Xia[1], Meiqi Feng[1], Miaomiao Zhang[2], Xiaoyan Zhang[1,2*] and Jianqing Xu[1,2*]

[1] Clinical Center of Biotherapy, Zhongshan Hospital & Institutes of Biomedical Sciences, Fudan University; Shanghai, P. R. China

[2] Shanghai Public Health Clinical Center & Institutes of Biomedical Sciences, Fudan University, Shanghai, P. R. China

[3]Bio-therapeutic Center, National Clinical Research Center for Infectious Disease, Shenzhen Third People's Hospital; The Second Hospital Affiliated with the School of Medicine, Southern University of Science and Technology, Shenzhen, Guangdong, China

*Corresponding authors: Jianqing Xu, xujianqing@fudan.edu.cn; Xiaoyan Zhang, zhangxiaoyan@fudan.edu.cn;

Description of Supplementary Information Files

Figure S1. Analysis of RABV-LT antigen expression by Western blot

Figure S2. Analysis of RABV-G-LT antigen expression by Western blot corresponds to the images presented in Figure 2B.

Figure S3. Gating strategy and representative flow cytometry profiles of cytokines secreted splenocytes cells in mice, as presented in Fig. 1 or Fig. 2.

Table S1. HLA Class I and Class II supertypes and their representative HLA Class I or Class II alleles.

Table S2. RABV-LT peptides used in the study

Table S3. RABV-G peptides used in the study

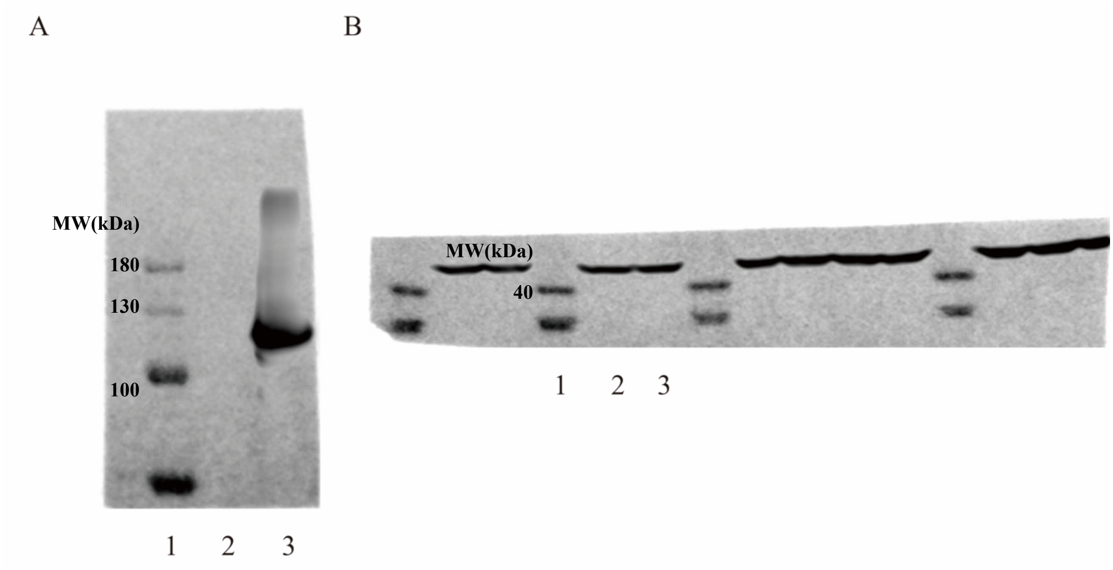

Figure S1. Analysis of RABV-LT antigen expression by Western blot corresponds to the images presented in Figure 1C. (A) Original Western blot image showing RABV-LT expression in HEK293T cells transfected with mRNA-LT, detected using Flag tag antibody. **(B)** Original Western blot image of β-actin corresponds to the samples of the lanes in Figure S1A. Lane 1: Thermo Scientific Page Ruler^{TM} Prestained Protein Ladder (No. 26616); Lane 2: Blank lysates; Lane 3: mRNA-RABV-LT lysates.

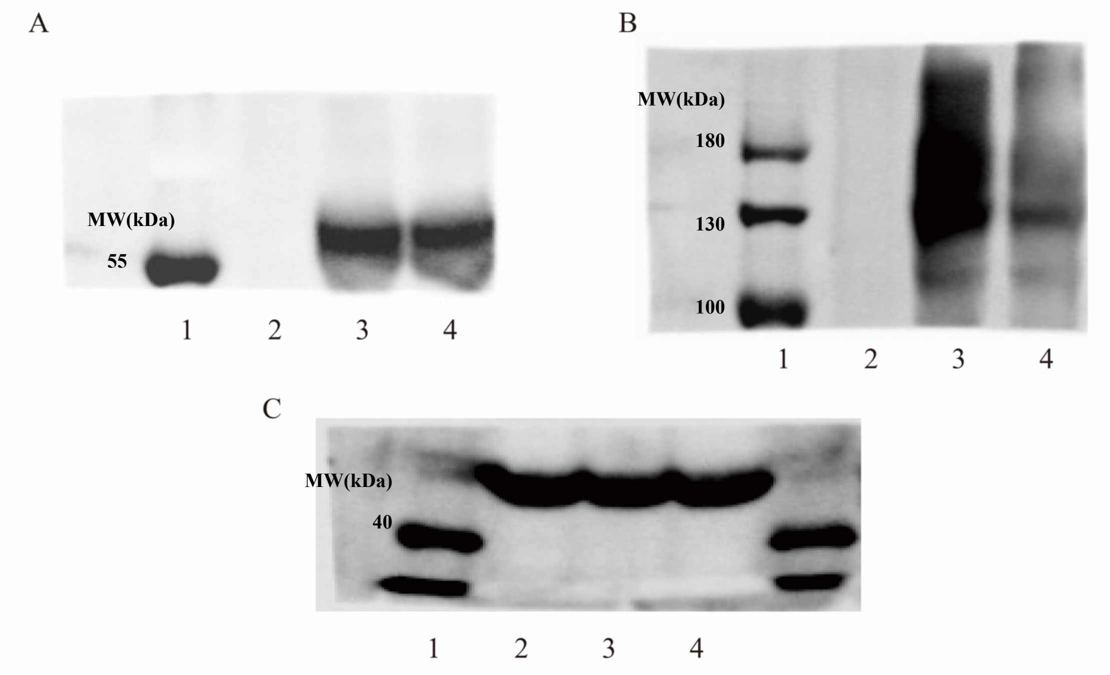

Figure S2. Analysis of RABV-G-LT antigen expression by Western blot corresponds to the images presented in Figure 2B. (A) Original Western blot image showing the RABV-G expression in HEK293T cells transfected with mRNA-RABV-G-LT (detected using anti-RABV-G antibody). (B) Original Western blot image showing RABV-LT expression in HEK293T cells transfected with mRNA-RABV-G-LT (detected using Flag tag antibody). (C) Western blot image of β-actin corresponds to the samples of the lanes in Figure S2A, S2B. Lane 1: Thermo Scientific Page Ruler^{TM} Prestained Protein Ladder (No. 26616); Lane 2: Blank lysates; Lane 3: mRNA-RABV-G-LT + 50 μM TMP lysates; Lane 4: mRNA-RABV-G-LT lysates.

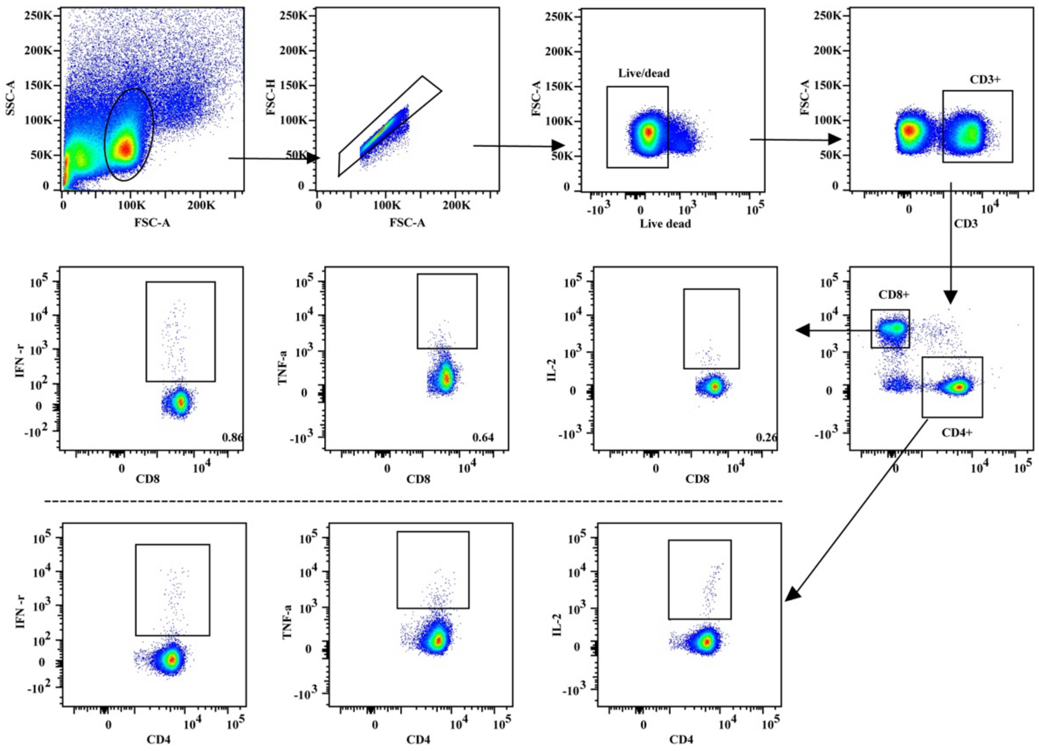

Figure S3. Gating strategy and representative flow cytometry profiles of cytokines secreted splenocytes cells in mice, as presented in Fig. 1 or Fig. 2. Cytokine + CD4+ T cells and Cytokine + CD8+ T cells ex vivo were stimulated with RABV-G peptide pools (1 μg/mL) or RABV-LT peptide pools (1 μg/mL) for 6 hours.

Table S1. HLA Class I and Class II supertypes and their representative HLA Class I or Class II alleles.

HLA-I supertype representative alleles	HLA-II supertype representative alleles	
HLA-A*01:01 (A1)	DRB1*0101	DRB1*1601
HLA-A*02:01 (A2)	DRB1*0102	DRB3*0101
HLA-A*03:01 (A3)	DRB1*0103	DRB3*0202
HLA-A*24:02 (A24)	DRB1*0301	DRB4*0101
HLA-A*26:01 (A26)	DRB1*0305	DRB4*0103
HLA-B*07:02 (B7)	DRB1*0401	DRB5*0101
HLA-B*08:01 (B8)	DRB1*0402	DRB5*0202
HLA-B*27:05 (B27)	DRB1*0403	DPA1*0103-DPB1*0201
HLA-B*39:01 (B39)	DRB1*0404	DPA1*0103-DPB1*0301
HLA-B*40:01 (B44)	DRB1*0405	DPA1*0103-DPB1*0401
HLA-B*58:01 (B58)	DRB1*0408	DPA1*0103-DPB1*0402
HLA-B*15:01 (B62)	DRB1*0701	DPA1*0103-DPB1*0601
	DRB1*0801	DPA1*0103-DPB1*1101
	DRB1*0803	DPA1*0103-DPB1*1701
	DRB1*0901	DPA1*0103-DPB1*2001
	DRB1*1001	DPA1*0103-DPB1*2301
	DRB1*1101	DQA1*0102-DQB1*0501
	DRB1*1104	DQA1*0102-DQB1*0602
	DRB1*1201	DQA1*0102-DQB1*0604
	DRB1*1301	DQA1*0103-DQB1*0501
	DRB1*1302	DQA1*0103-DQB1*0603
	DRB1*1303	DQA1*0201-DQB1*0201
	DRB1*1401	DQA1*0201-DQB1*0202
	DRB1*1402	DQA1*0401-DQB1*0301
	DRB1*1454	DQA1*0501-DQB1*0201
	DRB1*1501	DQA1*0501-DQB1*0301
	DRB1*1503	DQA1*0505-DQB1*0301

Table S2 RABV-LT peptides used in the study.

Number	Sequence	Number	Sequence	Number	Sequence
1	LMLYAQTWTSELVQR	29	VLRYEREAFSDFRSV	57	QKQVNMSYDLIICDE
2	AQTWTSELVQRDTRH	30	EREAFSDFRSVKMTY	58	NMSYDLIICDEVTDI
3	TSELVQRDTRHIGSA	31	FSDFRSVKMTYLTLM	59	DLIICDEVTDIASIN
4	VQRDTRHIGSAQGLL	32	RSVKMTYLTLMTYQS	60	CDEVTDIASINRITL
5	TRHIGSAQGLLYSIL	33	MTYLTLMTYQSHLLL	61	TDIASINRITLLMSD
6	GSAQGLLYSILVAIH	34	TLMTYQSHLLLQRVE	62	SINRITLLMSDFALS
7	GLLYSILVAIHDSGY	35	YQSHLLLQRVERMRA	63	ITLLMSDFALSIDGP
8	SILVAIHDSGYGTIF	36	LLLQRVERMRATLRQ	64	MSDFALSIDGPLYLV
9	AIHDSGYGTIFPVNI	37	RVERMRATLRQMSSL	65	ALSIDGPLYLVFKTY
10	SGYGTIFPVNIYILI	38	MRATLRQMSSLMRQV	66	DGPLYLVFKTYGTML
11	TIFPVNIYILIGSSI	39	LRQMSSLMRQVLGGH	67	YLVFKTYGTMLVNPD
12	VNIYILIGSSICFRP	40	SSLMRQVLGGHALSK	68	KTYGTMLVNPDYKAI
13	ILIGSSICFRPLELI	41	RQVLGGHALSKRFQN	69	TMLVNPDYKAIRAFP
14	SSICFRPLELISGVI	42	GGHALSKRFQNPLIS	70	NPDYKAIRAFPSVTG
15	FRPLELISGVISYIL	43	LSKRFQNPLISGLRV	71	KAIRAFPSVTGFVTQ
16	ELISGVISYILLRLD	44	FQNPLISGLRVRAVL	72	AFPSVTGFVTQVTSS
17	GVISYILLRLDNHPS	45	LISGLRVRAVLNMFP	73	VTGFVTQVTSSFSSE
18	YILLRLDNHPSLYIM	46	LRVRAVLNMFPDSKL	74	VTQVTSSFSSELYLR
19	RLDNHPSLYIMLREP	47	AVLNMFPDSKLVFNS	75	TSSFSSELYLRFSKR
20	HPSLYIMLREPSLRG	48	MFPDSKLVFNSLLVN	76	SSELYLRFSKRGKFF
21	YIMLREPSLRGEIFS	49	SKLVFNSLLVNDLMA	77	YLRFSKRGKFFRDAE
22	REPSLRGEIFSIPQK	50	FNSLLVNDLMASGTH	78	SKRGKFFRDAEYLTS
23	LRGEIFSIPQKREGN	51	LVNDLMASGTHPLPP	79	KFFRDAEYLTSSTLR
24	IFSIPQKREGNRSIL	52	LMASGTHPLPPSAIR	80	DAEYLTSSTLREMSL
25	PQKREGNRSILCYLQ	53	GTHPLPPSAIRYFQS	81	LTSSTLREMSLVLFN
26	EGNRSILCYLQHVLR	54	LPPSAIRYFQSVQKQ	82	TLREMSLVLFNCSSK
27	SILCYLQHVLRYERE	55	AIRYFQSVQKQVNMS	83	MSLVLFNCSSKSEMQ
28	YLQHVLRYEREAFS	56	FQSVQKQVNMSYDLI	84	LFNCSSKSEMQRARS
				85	SSKSEMQRARSLNYQ

Table S3 RABV-G peptides used in the study.

Number	Sequence	Number	Sequence
1	SPIDIHHLSCP	15	GFGKAYTIFNKTLME
2	TYTNFVGYV	16	AYTIFNKTL
3	PRYEESLHNPYPDYH	17	HYKSVRTWNEI
4	ESLHNPYPDYHWLRT	18	LKVGGRCHPHVNGVF
5	NPYPDYHWL	19	RCHPHVNGVFF
6	NPYPDYHWLRTVRTT	20	HVNGVFFNGIILGPD
7	PDYHWLRTVRTTKES	21	HVLIPEMQSSLLQQH
8	LRTVRTTKESLIIIS	22	SSVIPLMHPLADPST
9	RTTKESLIIISPSVT	23	LPNWGKYV L
10	AMQTSDETKWCPPDQ	24	KYVLMTAGAMI
11	CPPDQLVNL	25	GAMIGLVLIFSLMTW
12	PDQLVNLHDFRSDEI	26	RRANRPESKQRSFGG
13	DALESIMTTKSVSFR	27	FGGTGRNVSVTSQSG
14	KSVSFRRLSHLRKLV	28	IPSWESYKSGGQTRL
