# Supplementary material for: Rabies virus large protein-derived T-cell immunogen facilitates rapid viral clearance and enhances protection against lethal challenge in mice
Source: Commun Med (Lond). 2025 Apr 18;5:127. doi: 10.1038/s43856-025-00851-5 (PMC12008279; doi:10.1038/s43856-025-00851-5)
Supplement: Supplementary file 3 — Description of Additional Supplementary File [file 43856_2025_851_MOESM3_ESM.pdf]

Description of additional supplementary file

File name: Supplementary Data 1
Description: Data used to generate Figure 1

File name: Supplementary Data 2
Description: Data used to generate Figure 2

File name: Supplementary Data 3
Description: Data used to generate Figure 3

File name: Supplementary Data 4
Description: Data used to generate Figure 4

File name: Supplementary Data 5
Description: The distribution of high-affinity epitopes on the large protein among rabies virus(MHC I, MHC II_result)

File name: Supplementary Data 6
Description: Amino acid conservation rate in the L proteins among five virus strains
