# Supplementary material for: Rabies virus large protein-derived T-cell immunogen facilitates rapid viral clearance and enhances protection against lethal challenge in mice
Source: Commun Med (Lond). 2025 Apr 18;5:127. doi: 10.1038/s43856-025-00851-5 (PMC12008279; doi:10.1038/s43856-025-00851-5)
Supplement: Supplementary file 10 — Reporting-summary [file 43856_2025_851_MOESM10_ESM.pdf]

nature portfolio

Corresponding author(s): COMMSMED-24-0436-T

Last updated by author(s): October 25, 2024

Reporting Summary

Nature Portfolio wishes to improve the reproducibility of the work that we publish. This form provides structure for consistency and transparency in reporting. For further information on Nature Portfolio policies, see our Editorial Policies and the Editorial Policy Checklist.

Statistics

For all statistical analyses, confirm that the following items are present in the figure legend, table legend, main text, or Methods section.

n/a	Confirmed	
☐	☒	The exact sample size (n) for each experimental group/condition, given as a discrete number and unit of measurement
☐	☒	A statement on whether measurements were taken from distinct samples or whether the same sample was measured repeatedly
☐	☒	The statistical test(s) used AND whether they are one- or two-sided<br>*Only common tests should be described solely by name; describe more complex techniques in the Methods section.*
☒	☐	A description of all covariates tested
☒	☐	A description of any assumptions or corrections, such as tests of normality and adjustment for multiple comparisons
☐	☒	A full description of the statistical parameters including central tendency (e.g. means) or other basic estimates (e.g. regression coefficient) AND variation (e.g. standard deviation) or associated estimates of uncertainty (e.g. confidence intervals)
☐	☒	For null hypothesis testing, the test statistic (e.g. F, t, r) with confidence intervals, effect sizes, degrees of freedom and P value noted<br>*Give P values as exact values whenever suitable.*
☒	☐	For Bayesian analysis, information on the choice of priors and Markov chain Monte Carlo settings
☒	☐	For hierarchical and complex designs, identification of the appropriate level for tests and full reporting of outcomes
☒	☐	Estimates of effect sizes (e.g. Cohen's d, Pearson's r), indicating how they were calculated

Our web collection on statistics for biologists contains articles on many of the points above.

Software and code

Policy information about availability of computer code

Data collection	Data collection for flow cytometry assay using BD LSRFortessa™ Cell Analyzer (BD Biosciences). Enzyme-linked immunosorbent spot (ELISPOT) assay was performed with BioSpot plate reader (ChampSpot 437III; Beijing Sage Creation Science Co., Ltd.). One-step reverse transcription–polymerase chain reaction (RT-PCR) was carried out using the HiScript II One-Step qRT-PCR SYBR Green Kit (Vazyme) and performed with BIOER FQD-96A.
Data analysis	GraphPad Prism (v9.4.0), FlowJo (v10.4.0), NetMHC pan 4.1 servers (https://services.healthtech.dtu.dk/services/NetMHCpan-4.1/), NetMHCII pan 4.0 servers (https://services.healthtech.dtu.dk/services/NetMHCIIpan-4.0/), Clustal Omega servers (https://www.ebi.ac.uk/jdispatcher/msa/clustalo).

For manuscripts utilizing custom algorithms or software that are central to the research but not yet described in published literature, software must be made available to editors and reviewers. We strongly encourage code deposition in a community repository (e.g. GitHub). See the Nature Portfolio guidelines for submitting code & software for further information.

Data

Policy information about availability of data

All manuscripts must include a data availability statement. This statement should provide the following information, where applicable:

- Accession codes, unique identifiers, or web links for publicly available datasets
- A description of any restrictions on data availability
- For clinical datasets or third party data, please ensure that the statement adheres to our policy

The data generated and analyzed in this study are provided in the source data files. T cell immunogens were designed from the following five virus strains: PM1503 (accession number: DQ099525), CTN-1 (accession number: FJ959397), aG (accession number: GQ412744), ERA (accession number: EF206707), and CVS-11 (accession number: GQ918139). All other data in the study are available from the corresponding author upon reasonable request.

Human research participants

Policy information about studies involving human research participants and Sex and Gender in Research.

Reporting on sex and gender	*Use the terms sex (biological attribute) and gender (shaped by social and cultural circumstances) carefully in order to avoid confusing both terms. Indicate if findings apply to only one sex or gender; describe whether sex and gender were considered in study design whether sex and/or gender was determined based on self-reporting or assigned and methods used. Provide in the source data disaggregated sex and gender data where this information has been collected, and consent has been obtained for sharing of individual-level data; provide overall numbers in this Reporting Summary. Please state if this information has not been collected. Report sex- and gender-based analyses where performed, justify reasons for lack of sex- and gender-based analysis.*
Population characteristics	*Describe the covariate-relevant population characteristics of the human research participants (e.g. age, genotypic information, past and current diagnosis and treatment categories). If you filled out the behavioural & social sciences study design questions and have nothing to add here, write "See above."*
Recruitment	*Describe how participants were recruited. Outline any potential self-selection bias or other biases that may be present and how these are likely to impact results.*
Ethics oversight	*Identify the organization(s) that approved the study protocol.*

Note that full information on the approval of the study protocol must also be provided in the manuscript.

Field-specific reporting

Please select the one below that is the best fit for your research. If you are not sure, read the appropriate sections before making your selection.

☒ Life sciences ☐ Behavioural & social sciences ☐ Ecological, evolutionary & environmental sciences

For a reference copy of the document with all sections, see nature.com/documents/nr-reporting-summary-flat.pdf

Life sciences study design

All studies must disclose on these points even when the disclosure is negative.

Sample size	In compliance with laboratory animal ethics principles, sample sizes were not predetermined based on statistical methods but were chosen according to the standards of the field (at least three independent biological replicates), which could generate statistical significance for analysis.
Data exclusions	No data was excluded from studies.
Replication	Experimental assays were carried out in duplicate (or more) in compliance with or above the field's standards. Every replication attempt was successful.
Randomization	Throughout the whole experiment, samples and animals were randomized into groups.
Blinding	The investigators conducted the examination of lung pathology blindly. All the other data collected was quantifiable and blinding would not change any bias in data collected.

Reporting for specific materials, systems and methods

We require information from authors about some types of materials, experimental systems and methods used in many studies. Here, indicate whether each material, system or method listed is relevant to your study. If you are not sure if a list item applies to your research, read the appropriate section before selecting a response.

Materials & experimental systems

n/a	Involved in the study
☐	☒ Antibodies
☐	☒ Eukaryotic cell lines
☒	☐ Palaeontology and archaeology
☐	☒ Animals and other organisms
☒	☐ Clinical data
☒	☐ Dual use research of concern

Methods

n/a	Involved in the study
☒	☐ ChIP-seq
☐	☒ Flow cytometry
☒	☐ MRI-based neuroimaging

Antibodies

Antibodies used

1.Peroxidase AffiniPure Goat Anti-Mouse IgG(H+L) (Supplier: Yeasen, Cat#: 33201ES60), ELISA: 1:5000 dilution
2.streptavidin-conjugated HRP (Supplier: Yeasen, Cat#: 35105ES60), ELISA: 1:5000 dilution
3.Goat Anti-Mouse IgG1 heavy chain (Biotin) (Supplier: Abcam, Cat#: ab97238), ELISA: 1:5000 dilution
4.Goat Anti-Mouse IgG2a heavy chain (Biotin) (Supplier: Abcam, Cat#: ab97243), ELISA: 1:5000 dilution
5.Goat Anti-Mouse IgG2b heavy chain (Biotin) (Supplier: Abcam, Cat#: ab97248), ELISA: 1:5000 dilution
6.HRP-conjugated anti-monkey lgG antibodies (Supplier: lnvitrogen, Cat#: PAl-84631), ELISA: 1:5000 dilution
7.anti-RABV-G (glycoprotein) protein antibody, Rabies Virus Antibody (Rab-50) (Supplier: Santa Cruz, Cat#: sc-57994), Western blot: 1:1000 dilution
8.anti-CD3-Percpcy5.5 (Supplier: BioLegend, Cat#: 100218, clone: 17A2), flow cytometry: 1μg /test, 1:20 dilution
9.anti-CD8-FITC (Supplier: BioLegend, Cat#: 100706, clone: 53-6.7), flow cytometry: 1μg /test, 1:50 dilution
10.anti-CD4-AF700 (Supplier: BD Biosciences, Cat#: 557956, clone: RM4-5), flow cytometry: 0.5 μg /test, 1:80 dilution
11.anti-IL-2-APC (Supplier: BioLegend, Cat#: 503810, clone: JES6-5H4), flow cytometry: 0.25 μg /test, 1:100 dilution
12.anti-TNF-α-BV605 (Supplier: BioLegend, Cat#: 506329, clone: MP6-XT22), flow cytometry: 0.5 μg /test, 1:80 dilution
13.anti-IFN-γ-PE (Supplier: BD Biosciences, Cat#: 554412, clone: XMGl.2), flow cytometry: 0.5 μg /test, 1:80 dilution
14.ANTI-FLAG M2 antibody (Supplier: Sigma–Aldrich, Cat#: F3165, clone: clone M2), Western blot: 1:2000 dilution

Validation

All antibodies are commercially available and were commercially validated.
1. https://www.yeasen.com/products/detail/407
2. https://www.yeasen.com/products/detail/281
3. https://www.abcam.com/products/secondary-antibodies/goat-mouse-igg1-heavy-chain-biotin-ab97238.html
4. https://www.abcam.com/products/secondary-antibodies/goat-mouse-igg2a-heavy-chain-biotin-ab97243.html
5. https://www.abcam.com/products/secondary-antibodies/goat-mouse-igg2b-heavy-chain-biotin-ab97248.html
6. https://www.thermofisher.cn/cn/zh/antibody/product/Goat-anti-Monkey-IgG-H-L-Secondary-Antibody-Polyclonal/PA1-84631
7. https://www.scbt.com/p/rabies-virus-antibody-rab-50
8. https://www.biolegend.com/fr-ch/products/percp-cyanine5-5-anti-mouse-cd3-antibody-5596
9. https://www.biolegend.com/fr-ch/products/fitc-anti-mouse-cd8a-antibody-153
10. https://www.bdbiosciences.com/en-us/products/reagents/flow-cytometry-reagents/research-reagents/single-color-antibodies-ruo/alexa-fluor-700-rat-anti-mouse-cd4.557956
11. https://www.biolegend.com/fr-ch/products/apc-anti-mouse-il-2-antibody-950
12. https://www.biolegend.com/fr-ch/products/brilliant-violet-605-anti-mouse-tnf-alpha-antibody-7682
13. https://www.bdbiosciences.com/en-us/products/reagents/flow-cytometry-reagents/research-reagents/single-color-antibodies-ruo/pe-rat-anti-mouse-ifn.554412
14. https://www.sigmaaldrich.cn/CN/zh/product/sigma/f3165

Eukaryotic cell lines

Policy information about cell lines and Sex and Gender in Research

Cell line source(s)	HEK293T cells (Cat. No# CRL-3216) were from ATCC; BHK-21 cells were kindly provided by Dr. Zhang Shuye at Fudan University.
Authentication	None of the cell lines used were authenticated.
Mycoplasma contamination	All cell lines were negative for mycoplasma contamination.
Commonly misidentified lines (See ICLAC register)	No commonly misidentified lines were used.

Animals and other research organisms

Policy information about studies involving animals; ARRIVE guidelines recommended for reporting animal research, and Sex and Gender in Research

Laboratory animals

Female BALB/c mice (6–8 weeks old, specific-pathogen-free) were purchased from Suzhou Hua Chang Biological Co., Ltd. Female

Laboratory animals	rhesus macaques (approximately three years old) obtained from a domestic source (Ningbo, China) were all housed in the Shanghai Public Health Clinical Center (SPHCC) animal facility.
Wild animals	The study did not involve wild animals.
Reporting on sex	Female mice and female rhesus macaques were used in this study. Sex was not considered in study design.
Field-collected samples	The study did not involve samples collected from the field.
Ethics oversight	The animal experiments were conducted according to the recommendations of the SPHCC Guide for the Care and Use of Laboratory Animals.

Note that full information on the approval of the study protocol must also be provided in the manuscript.

Flow Cytometry

Plots

Confirm that:

☒ The axis labels state the marker and fluorochrome used (e.g. CD4-FITC).

☒ The axis scales are clearly visible. Include numbers along axes only for bottom left plot of group (a 'group' is an analysis of identical markers).

☒ All plots are contour plots with outliers or pseudocolor plots.

☒ A numerical value for number of cells or percentage (with statistics) is provided.

Methodology

Sample preparation	The spleens were collected from immunized animals. After euthanizing the mice humanely, the bodies were soaked in 75% alcohol and then dried with absorbent paper. The mice were placed in a biosafety cabinet, lying on their left side. Using scissors, the skin was cut open to reveal the deep red spleen. The spleen was gently removed with forceps and placed in a centrifuge tube containing 4 mL of R10 medium. The spleen was then placed in a 40 μm filter soaked in R10 medium. The spleen was ground with the plunger of a 10 mL syringe, the suspension was aspirated and transferred to a 15 mL centrifuge tube. Centrifugation was carried out at 800 g for 3 min, the supernatant was discarded, and the cell pellet was retained. The cell pellet was treated with 3 mL of red blood cell lysis buffer for 5-8 min, followed by the addition of 5 mL of R10 complete medium to terminate the lysis reaction. The tube was centrifuged again at 800 g for 3 min, the supernatant was discarded, and the cell pellet was resuspended in 5 mL of R10 medium. The spleen cells were then counted and typically diluted 25-fold. The cell concentration was adjusted to 1E6/mL based on the cell count, and flow cytometric analysis was performed.
Instrument	BD LSRFortessa™ Cell Analyzer (BD Biosciences)
Software	FlowJo software (v10.4.0)
Cell population abundance	No sorting experiment were applied in this study.
Gating strategy	The gating strategies are mainly described in Main text. For all experiments, initial cell populations were gated for a live population using FSC and SSC plot of cell only sample. The gate was set to remove cell debris (small FSC v SSC) and large clumps or aggregates of cells (large FSC or SSC) and used across all samples. Then FSC-H vs. FSC-A allowed for selection of "singlets", avoiding cells sticking to each other as doublets. "Viable/live" cells (Amcyan LIVE/DEAD Aqua negative) were then positively gated from the singlets. The specific RABV-LT- or RABV-G- positive CD4+ or CD8+ T cells were gated by live spleen cells from empty-LNP (negative control) immunized mice stained by anti- CD3- PerCP-Cyanine5.5, anti-CD8-FITC and anti-CD4-Alexa Fluor 700. The antigen specific IFN-γ or TNF-α or IL-2 producing T cells from immunized mice were gated from spleen cells with co-expression of CD3, CD8 /CD4.

☒ Tick this box to confirm that a figure exemplifying the gating strategy is provided in the Supplementary Information.
